# Technical note: Partitioning of gated single photon emission computed tomography raw data for protocols optimization

**DOI:** 10.1002/acm2.13508

**Published:** 2021-12-17

**Authors:** Cleiton Cavalcante Queiroz, Marcos Antonio Dorea Machado, Antonio Augusto Brito Ximenes, Andre Gustavo Silva Pino, Eduardo Martins Netto

**Affiliations:** ^1^ Department of Nuclear Medicine São Rafael Hospital/ Rede D'or Salvador Bahia Brazil; ^2^ Department of Nuclear Medicine Cardio Pulmonar Hospital/ Rede D'or Salvador Bahia Brazil; ^3^ Department of Nuclear Medicine Hospital Universitario Professor Alberto Antunes/Ebserh Maceio Alagoas Brazil; ^4^ Department of Health Technology Evaluation Complexo Hospitalar Universitário Prof. Edgard Santos/Ebserh Salvador Bahia Brazil; ^5^ Infectious Disease Research Laboratory Complexo Hospitalar Universitário Prof. Edgard Santos/Ebserh Universidade Federal da Bahia Salvador Bahia Brazil

**Keywords:** gated SPECT, low‐dose, optimization, raw data, shorter‐scan‐time

## Abstract

**Purpose:**

Methodologies for optimization of SPECT image acquisition can be challenging due to imaging throughput, physiological bias, and patient comfort constraints. We evaluated a vendor‐independent method for simulating lower count image acquisitions.

**Methods:**

We developed an algorithm that recombines the ECG‐gated raw data into reduced counting acquisitions. We then tested the algorithm to simulate reduction of counting statistics from phantom SPECT image acquisition, which was synchronized with an ECG simulator. The datasets were reconstructed with a resolution recovery algorithm and the summed stress score (SSS) was assessed by three readers (two experts and one automatic).

**Results:**

The algorithm generated varying counting levels, simulating multiple examinations at the same time. The error between the expected and the simulated countings ranged from approximately 5% to 10% for the ungated simulations and 0% for the gated simulations.

**Conclusions:**

The vendor‐independent algorithm successfully generated lower counting statistics datasets from single‐gated SPECT raw data. This method can be readily implemented for optimal SPECT research aiming to lower the injected activity and/ or to shorten the acquisition time.

## 1 INTRODUCTION

Single photon emission computed tomography (SPECT) is a widely used imaging method for the diagnosis and management of many diseases.^1^, ^2^ The method consists of injecting radiopharmaceuticals to map their distribution in an organ. Optimizing the imaging protocols to lower the amount of administered radiopharmaceutical activities to patients, and/ or to shorten the acquisition duration, is recommendable.^3^, ^4^, ^5^, ^6^, ^7^, ^8^, ^9^, ^10^

Advances in detectors, reconstruction software, and imaging protocols allow for the reduction of counting statistics (which is a function of the administered activity and the acquisition duration) without compromising the image quality.^4^, ^5^, ^6^, ^7^, ^8^, ^9^, ^10^, ^11^, ^12^, ^13^, ^14^, ^15^, ^16^, ^17^ However, defining the optimal counts requires to access various counting acquisition levels of the same patient.

Experimental schemes in image optimization studies play a key role in SPECT research. Physiological changes in patients between experiments, facility imaging throughput, and patient comfort are important research constraints. Most studies scanned the patient two times using a variety of acquisition durations^4^, ^5^, ^6^, ^7^, ^8^ or preferably used list‐mode acquisition.^9^, ^10^, ^11^, ^12^, ^13^ List‐mode enables the use of the same image dataset to simulate various scanning durations and/or injected activity. With SPECT, only a few systems allow for simulating multiple scan durations^9^, ^10^ or setting up multiple acquisition times concurrently.^17^, ^18^ Therefore, vendor‐independent methods that allow for nuclear medicine practioneers to easily simulate varying counting levels could facilitate the execution of protocol optimization studies.^14^, ^15^, ^16^

The gated SPECT (G‐SPECT) has been used in most clinical settings to assess the functional information (e.g., ejection fraction, wall motion, and thickness in cardiac studies).^19^ Since G‐SPECT employs a partitioning method that splits the acquisition data into subsets of defined number of frames, it could be a promising candidate to make lower counts simulations widely available.^14^

In this study, we used G‐SPECT to present a vendor‐independent method to simulate varying amounts of counting levels.

## 2 METHODS

In a typical ECG‐gated SPECT acquisition, the cardiac cycle is divided into 8 or 16 frames, with each frame corresponding to a specific phase of the cardiac cycle in the tomographic projection.^20^ The images acquired from the same frame are summated at each projection and subsequently reconstructed using all projections to generate the diagnostic image.

**FIGURE 1:**
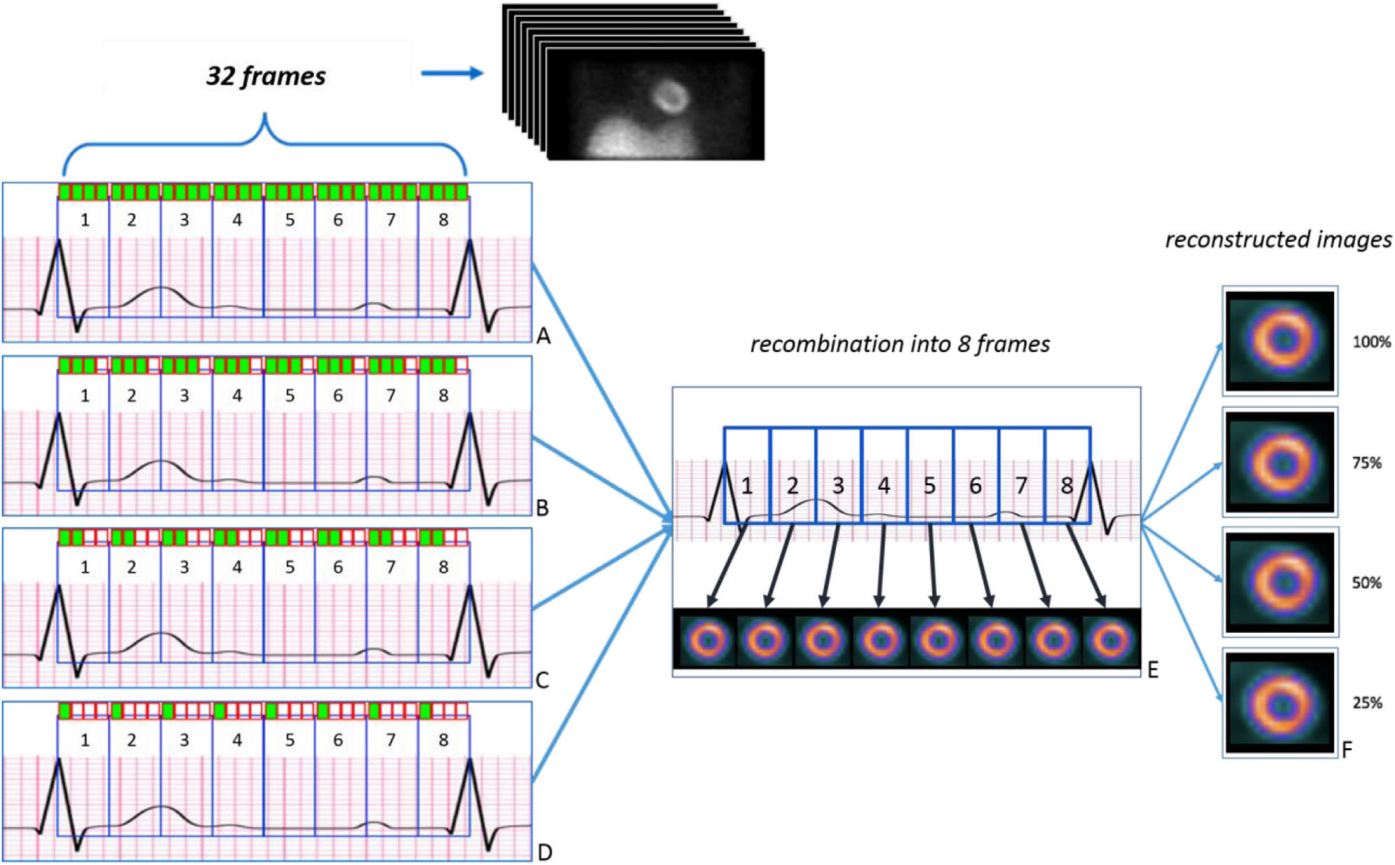
Partitioning and recombination of frames into lower counting statistics data. The colored marks indicate the selected frame to be rebinned into new simulated images. (A) 100% simulated statistics of the original data, 32 frames divided into 8 frames. (B) 75% simulated statistics of the original data, 24 frames divided into 8 frames. (C) 50% simulated statistics of the original data, 16 frames divided into 8 frames. (D) 25% simulated statistics of the original data, 8 frames divided into 8 frames. (E) Simulated dataset with reduced frames merged into its respective spot. (F) Reconstructed image of the summated frames

Our method consists of partitioning the cardiac cycle into ƞ frames using the gama‐camera workstation, and then recombining a subset of these frames to a required counting level using our software, forming a new image with eight frames. Figure 1 illustrates a cardiac cycle divided into ƞ = 32 frames. Following this, new simulated images can be reduced to 3/4 (Figure 1b), 1/2 (Figure 1c), 1/4 (Figure 1d), or recombined to the original ungated and gated images (Figure 1a).

An algorithm for implementation of the example in Figure 1e was developed in *Phyton* ^21^ (Supporting information 1). After input of the G‐SPECT image with ƞ frames, the algorithm generates new datasets with simulated 100%, 75%, 50%, and 25% countings of the ungated and the gated images.

Phantom image acquisition and reconstruction were used to analyze our algorithm. According to Table 1, image acquisitions were performed with three Siemens SPECT systems using either the static cardiac phantom ECT/TOR/P (Data Spectrum Corporation)^22^ or the PET/SPECT Phantom Source Tank (76‐823) with the Cardiac Insert (76‐825) (Fluke Biomedical). The image acquisitions were performed synchronized either with an ECG simulator (Cardiac Trigger Monitor 7600) or synchronized with the heart cycle of a healthy volunteer (C.Q.) (ƞ = 32 frames). Detailed imaging parameters are available at supporting information 2.

**TABLE 1:** Experimental schemes

Scheme	SPECT system	ECG simulation	Cardiac phantom
1	e.cam	Cardiac Trigger Monitor 7600	ECT/TOR/P
2	Symbia Evo	Cardiac Trigger Monitor 7600	76‐823 / 76–825
3	Symbia Intevo	Healthy volunteer	ECT/TOR/P

The image raw data were retrieved from the Siemens workstations and transferred to another workstation with the software, recombined into four datasets and then sent back to the Siemens workstation.

For each simulated image, the absolute error in the new datasets was estimated by calculating the percentage difference in the expected counting statistics, according to Equation (1):

(1) $$\%\mathrm{Diff}\;=\;100.\left(\frac{E-S}{E}\right),$$

where *E* corresponds to the theoretical expected counting statistics and *S* corresponds to the counting statistics of 64 summed projections in the simulated image.

Projections were reconstructed using the Flash3D™ resolution recovery algorithm and the summed stress score (SSS) (17‐segment polar map) was computed using Corridor4DM™ software (Michigan, United States).^23^ Each simulated dataset was scored manually by two experienced nuclear medicine physicians, and scored automatically with the Corridor4DM™. The mean and 95% confidence interval (CI) of SSS was assessed for each counting level.

## 3 RESULTS

The absolute error between the expected and the simulated counting statistics (Equation (1)) for schemes 1–3 is presented in Table 2.

**TABLE 2:** Absolute error %Diff

Scheme	Simulation	A	B	C	D
**1**	gated	0.00%	0.00%	0.00%	0.00%
**1**	ungated	4.96%	4.98%	5.02%	4.95%
**2**	gated	0.00%	0.00%	0.00%	0.00%
**2**	ungated	9.65%	9.66%	9.64%	9.68%
**3**	gated	0.00%	0.00%	0.00%	0.00%
**3**	ungated	9.60%	9.60%	9.54%	9.57%

*Note*: A–D: 100%, 75%, 50%, and 25% simulated counts, respectively.

G‐SPECT generates a standard ungated perfusion image by summating temporal frames together.^24^ The Siemens systems, however, generate two independent gated and ungated datasets with 5%–10% less counts in the gated dataset. Since our software used the gated image to generate the simulated ungated, the reduced counts in the original gated image was propagated to the simulated ungated image.

**FIGURE 2:**
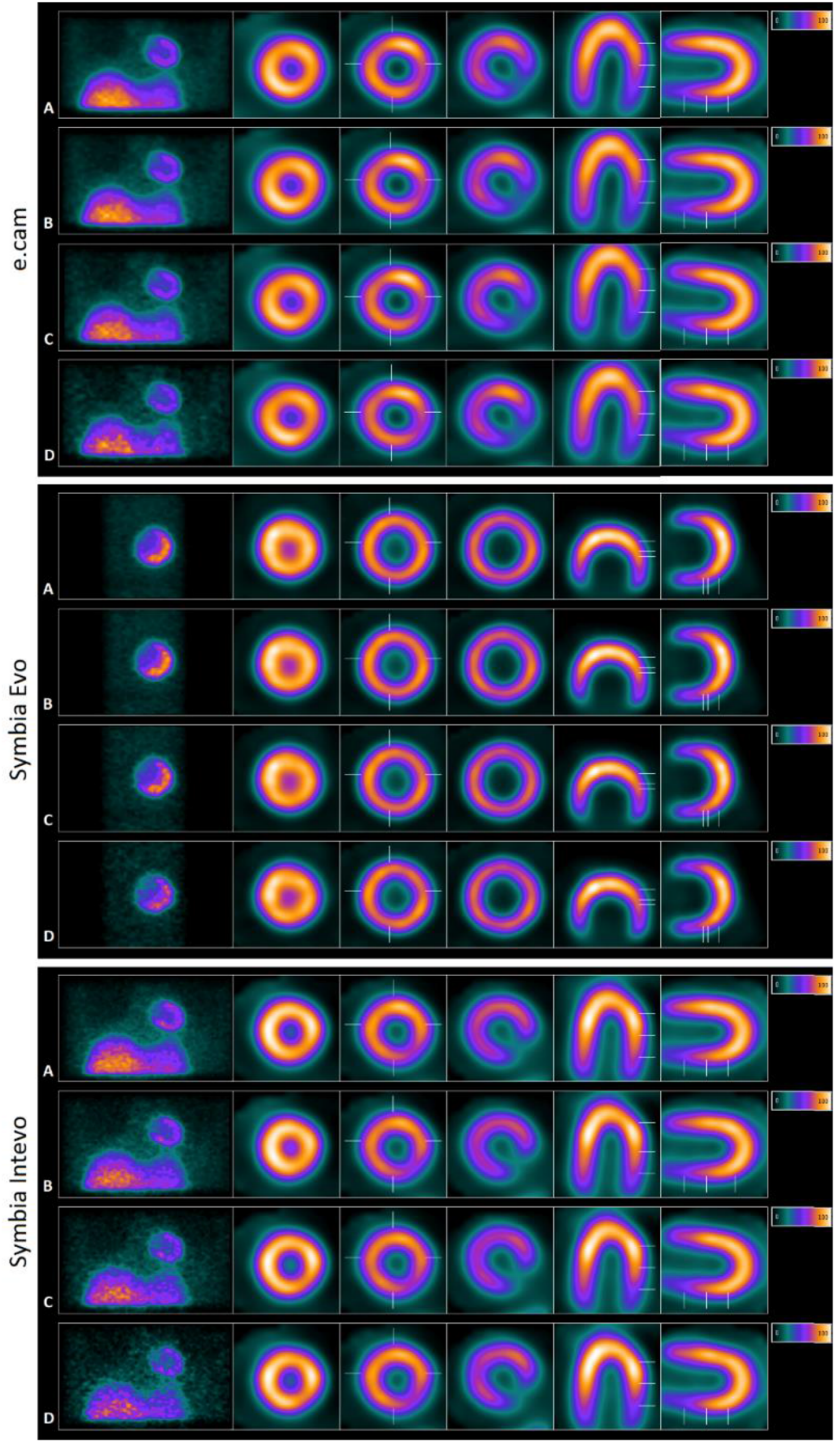
Simulated ungated projections (left) and reconstructed images (right). Simulated ungated imagens with 100% (A), 75% (B), 50% (C), and 25% (D) of the original raw data

**FIGURE 3:**
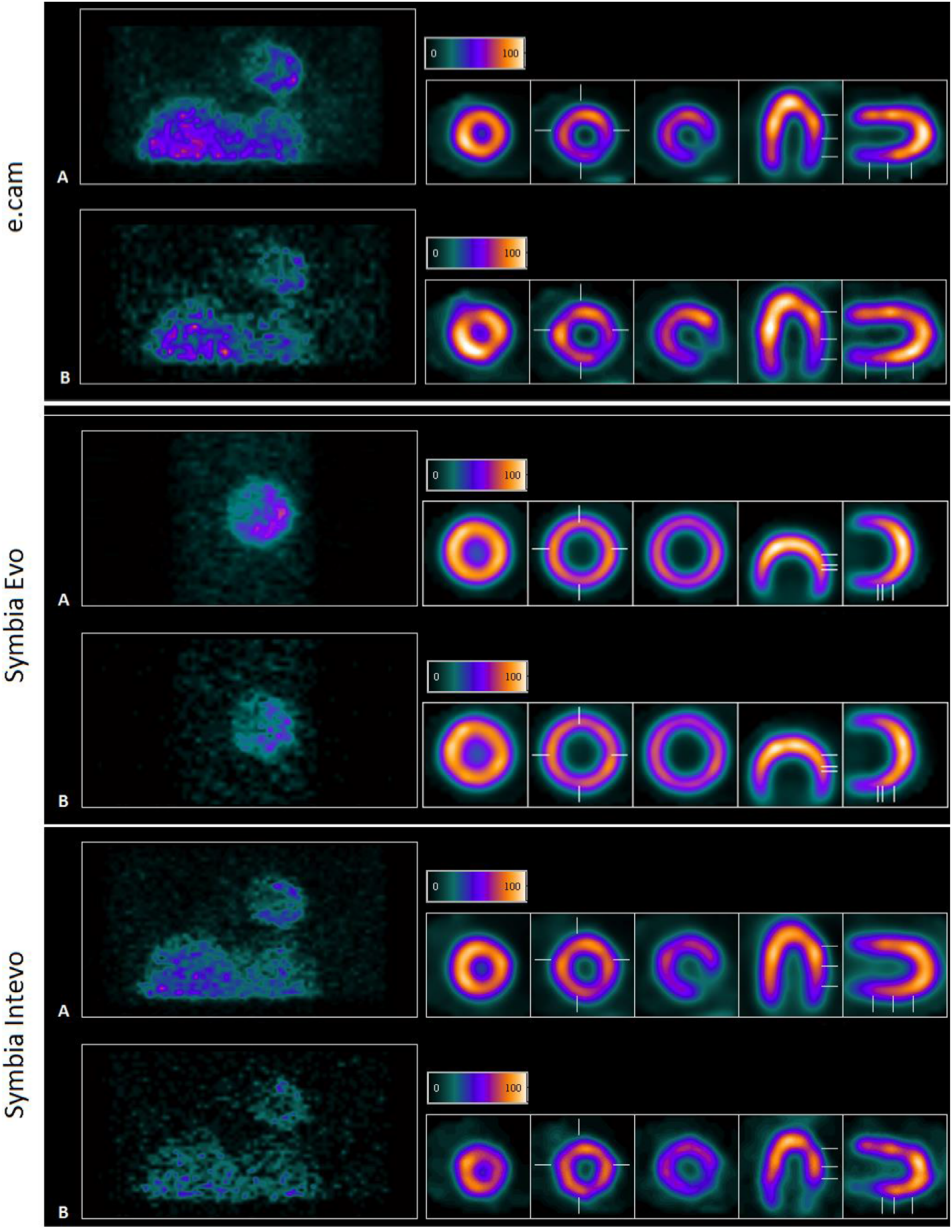
Gated projections (left) and reconstructed (right) images. Simulated statistics of the 8‐frames gated data with 100% (A) and 25% (B) counts of the original raw data

The left‐hand side of Figure 2 shows a projection of the partitioned/recombined raw data for the simulated ungated images. On the right is the reconstructed image showing the short, horizontal, and vertical axis of the phantom. Counting levels relative to the original input are shown in percentage terms as 100, 75, 50, and 25 from top to bottom. Figure 3 shows the simulated data for the gated image with 100% and 25% countings.

**FIGURE 4:**
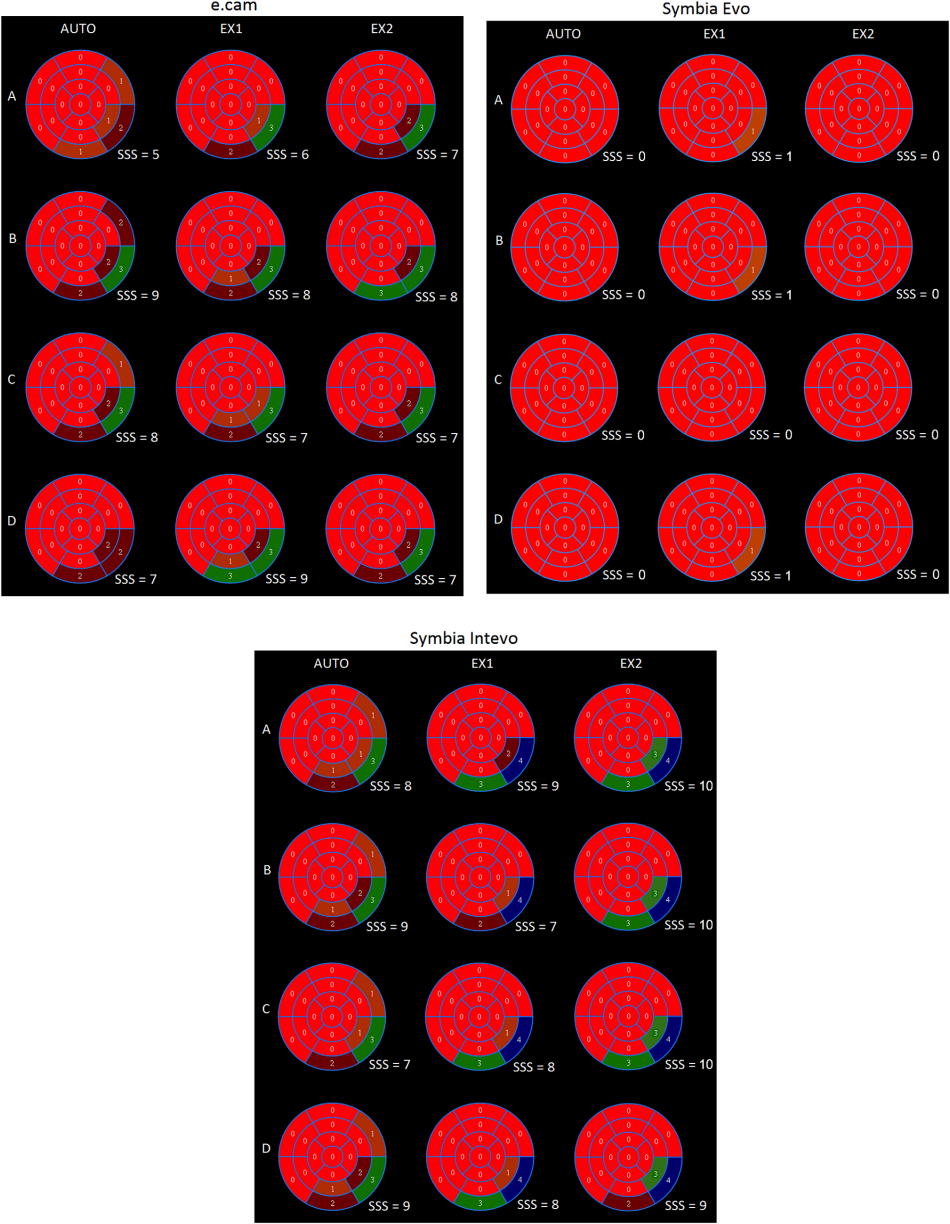
polar map quantifications for the simulated ungated images. (A–D) represents quantifications of the ungated simulations for 100%, 75%, 50%, and 25% of the original data statistics, respectively. AUTO: automatic quantification using standard database of normality. EX1 and EX2: nuclear medicine expert quantifications. SSS: summed stress score for 17‐segment polar map

Figure 4 presents the polar map with the SSS quantifications for the reconstructed ungated images. The mean SSS value over three observers for 100%, 75%, 50%, and 25% counting levels were, respectively, 6.0±2.0, 8.3±1.1, 7.3±1.1% and 7.3±3.0 for the e.cam system; 0.3±1.1, 0.3±1.1, 0.0±0.0% and 0.3±1.1 for the Symbia Evo; and 9.0±2.0, 8.7±3.0, 8.3±3.0, and 8.7±1.1 for the Symbia Intevo.

## 4 DISCUSSION

We developed and provided a vendor‐independent algorithm for raw data partitioning/ recombination of G‐SPECT into varying counting levels to adequately simulate lower injected activity (or shorter acquisition duration) of SPECT studies. The feasibility of our method was validated in a phantom experiment and successfully produced reliable results.

Assessment of image quality through clinical indices has been widely reported.^6^, ^7^, ^8^, ^9^, ^10^, ^11^, ^12^, ^22^ We showed a typical example of an optimization workflow to demonstrate the validity of our method. Experimental schemes resulted %Diff between 5% and 10%, suggesting older generation e.cam system yields slightly less counting loss in the gated image than current generation Symbia systems.

As expected, the reduction of the counting statistics did not significantly influence the SSS quantification. Lacchi and colleagues obtained similar trends at the same counting levels in a human model, where the SSS quantification was affected only for overweight and obese subjects.^13^ Here, we used a phantom that did not mimic the photon attenuation. Furthermore, we used a resolution recovery reconstruction algorithm, which is known to produce good image quality performance in various count levels.^4^, ^5^, ^6^, ^7^, ^8^, ^11^, ^13^, ^22^ In addition, the phantom model produces images from the static activity distributions, while a real patient model would have moving organs and thus lower quality images. Then, these may have improved the image quality over the lower counting levels seen in Figure 2.

In this study, we used ƞ = 32 frames which is the maximum setup available at the Siemens systems. Rebinning ƞ into 8 frames using our software results in a standard cardiac‐gated image and enables the recovery of perfusion and gated information. This application of our method is appropriate for the most complex human model, where capturing the information (ECG‐synchronized) of the moving organ is required. Moreover, any simpler rebinings can provide lower counting statistics for other clinical applications such as neurology and oncology.^4^ This example is also available (supporting information 1) to generate new datasets with 1/ ƞ rebinnings.

The ability to set up multiple acquisition times is not likely to be available in most commercial systems. We therefore provided a vendor‐independent algorithm which is readily applicable to different SPECT exams. This technique is easy to implement, able to be performed rapidly, and is highly robust. Our method improves the image optimization process since the patient is submitted to one only image acquisition, which increases patient comfort, while enhancing the routine throughput during optimization studies.

## CONFLICT OF INTEREST

The authors declare that there is no conflict of interest that could be perceived as prejudicing the impartiality of the research reported.

## AKNOWLEDGMENTS

We appreciate the support of Programa de Pós‐Graduação em Medicina e Saúde, Faculdade de Medicina da Bahia, Universidade Federal da Bahia, Salvador, Bahia, Brazil; and Coordenacão de Aperfeiçoamento de Pessoal de Nível Superior‐Brazil (CAPES)‐Finance Code 001. We also thank Bruno Cesar da Rocha Farias Santana for his contribution with the experiments and data collection.

## AUTHOR CONTRIBUTIONS

Cleiton C. Queiroz contributed to study design, data collection, algorithm development, data analysis, interpretation of results, and final approval of the version to be published. Marcos A. D. Machado contributed to study conception and design, data analysis and interpretation of results, drafting the article, and final approval of the version to be published. Antonio A. B. Ximenes contributed to data analysis, revising the article, and final approval of the version to be published. Andre G. S. Pino contributed to data analysis, revising the article, and final approval of the version to be published. Eduardo M. Netto contributed to interpretation of results, revising the article, and final approval of the version to be published.

## Supporting information

Supporting information

Supporting information

## References

[1] Ritt P , Vija H , Hornegger J , Kuwert T . Absolute quantification in SPECT. Eur J Nucl Med Mol Imaging. 2011;38(Suppl 1):S69‐S77. 21484383 10.1007/s00259-011-1770-8

[2] Mariani G , Bruselli L , Kuwert T , et al. A review on the clinical uses of SPECT/CT. Eur J Nucl Med Mol Imaging. 2010;37:1959‐1985. 20182712 10.1007/s00259-010-1390-8

[3] Menezes VO , Machado MAD , Queiroz CC , et al. How does Lean Six Sigma method improve healthcare practice in nuclear medicine departments? A successful case of dedicated software applications in oncological PET/CT. J Nucl Med. 2018;59(Suppl 1):1013.

[4] Zacho HD , Manresa JAB , Aleksyniene R , et al. Three‐minute SPECT/CT is sufficient for the assessment of bone metastasis as add‐on to planar bone scintigraphy: prospective head‐to‐head comparison to 11‐min SPECT/CT. EJNMMI Res. 2017;7(1):1‐7. 28058659 10.1186/s13550-016-0252-1 PMC5215994

[5] Valenta I , Treyer V , Husmann L , et al. New reconstruction algorithm allows shortened acquisition time for myocardial perfusion SPECT. Eur J Nucl Med Mol Imaging. 2010;37(4):750‐757. 19921186 10.1007/s00259-009-1300-0

[6] Borges‐Neto S , Pagnanelli RA , Shaw LK , et al. Clinical results of a novel wide beam reconstruction method for shortening scan time of Tc‐99m cardiac SPECT perfusion studies. J Nuc Cardiol. 2007;14(4 SPEC. ISS.):555‐565. 10.1016/j.nuclcard.2007.04.022 17679065

[7] DePuey EG , Gadiraju R , Clark J , Thompson L , Anstett F , Shwartz SC . Ordered subset expectation maximization and wide beam reconstruction “half‐time” gated myocardial perfusion SPECT functional imaging: a comparison to “full‐time” filtered backprojection. J Nuc Cardiol. 2008;15(4):547‐563. 10.1016/j.nuclcard.2008.02.035 18674723

[8] Druz RS , Phillips LM , Chugkowski M , Boutis L , Rutkin B , Katz S . Wide‐beam reconstruction half‐time SPECT improves diagnostic certainty and preserves normalcy and accuracy: a quantitative perfusion analysis. J Nuc Cardiol. 2011;18(1):52‐61. 10.1007/s12350-010-9304-5 21181520

[9] Nakazato R , Berman DS , Hayes SW , et al. Myocardial perfusion imaging with a solid‐state camera: simulation of a very low dose imaging protocol. J Nuc Med. 2013;54(3):373‐379. 10.2967/jnumed.112.110601 PMC3594528 23321457

[10] Palyo RJ , Sinusas AJ , Liu YH . High‐sensitivity and high‐resolution SPECT/CT systems provide substantial dose reduction without compromising quantitative precision for assessment of myocardial perfusion and function. J Nuc Med. 2016;57(6):893‐899. 10.2967/jnumed.115.164632 26848173

[11] Machado MAD , Menezes VO , Namías M , et al. Protocols for harmonized quantification and noise reduction in low‐dose oncologic 18F‐FDG PET/CT imaging. J Nuc Med Technol. 2019;47(1):47‐54. 10.2967/jnmt.118.213405 30076252

[12] Zhang J , Liu X , Knopp MI , Ramaswamy B , Knopp MV . How Long of a Dynamic 3′‐Deoxy‐3′‐[18 F] fluorothymidine ([18 F] FLT) PET acquisition is needed for robust kinetic analysis in breast cancer? Mol Imaging Biol. 2019;21(2):382‐390. 29987617 10.1007/s11307-018-1231-x PMC7201384

[13] Menezes VO , Machado MAD , Queiroz CC , et al. Optimization of oncological 18F‐FDG PET/CT imaging based on a multiparameter analysis. Med Phys. 2016;43(2):930‐938. https://aapm.onlinelibrary.wiley.com/doi/10.1118/1.4940354 26843253 10.1118/1.4940354

[14] Armstrong IS , Arumugam P , James JM , Tonge CM , Lawson RS . Reduced‐count myocardial perfusion SPECT with resolution recovery. Nucl Med Commun. 2012;33(2):121‐129. 22107994 10.1097/MNM.0b013e32834e10d5

[15] Kim IH , Lee SJ , An YS , Choi SY , Yoon JK . Simulating dose reduction for myocardial perfusion SPECT using a Poisson resampling method. Nucl Med Mol Imaging. 2021;55(5):245‐252. 10.1007/s13139-021-00710-w 34721717 PMC8517053

[16] Lawson RS , White D , Nijran K , et al. An audit of half‐count myocardial perfusion imaging using resolution recovery software. Nucl Med Commun. 2014;35(5):511‐521. 24448215 10.1097/MNM.0000000000000078

[17] Lecchi M , Martinelli I , Zoccarato O , Maioli C , Lucignani G , Del Sole A . Comparative analysis of full‐time, half‐time, and quarter‐time myocardial ECG‐gated SPECT quantification in normal‐weight and overweight patients. J Nucl Cardiol. 2017;24(3):876‐887. 26911365 10.1007/s12350-015-0382-2

[18] Coles DE , Murray D , Bertelsen H. Multiple simultaneous acquisition of gamma camera data sets. US patent 6,900,441. May 31, 2005.

[19] Paul AK , Nabi HA . Gated myocardial perfusion SPECT: basic principles, technical aspects, and clinical applications. J Nucl Med Technol. 2004;32(4):179‐187. 15576339

[20] Montelatici G , Sciagrà R , Passeri A , et al. Is 16‐frame really superior to 8‐frame gated SPECT for the assessment of left ventricular volumes and ejection fraction? Comparison of two simultaneously acquired gated SPECT studies. Eur J Nucl Med Mol Imaging. 2008;35:2059‐2065. 18648807 10.1007/s00259-008-0866-2

[21] Van Rossum G , Drake FL . Python 3 Reference Manual. CreateSpace; 2009.

[22] Zoccarato O , Scabbio C , Ponti E , et al. Comparative analysis of iterative reconstruction algorithms with resolution recovery for cardiac SPECT studies. A multi‐center phantom study. J Nucl Cardiol. 2014:135‐148. 24272971 10.1007/s12350-013-9821-0

[23] Ficaro EP , Lee BC , Kritzman JN , et al. Corridor4DM: the Michigan method for quantitative nuclear cardiology. J Nucl Cardiol. 2007;14:455‐465. 17679053 10.1016/j.nuclcard.2007.06.006

[24] Paul AK , Nabi HA . Gated myocardial perfusion SPECT: basic principles, technical aspects, and clinical applications. J Nucl Med Technol. 2004;32(4):179‐187. 15576339

