# Supplementary material for: Technical note: Partitioning of gated single photon emission computed tomography raw data for protocols optimization
Source: J Appl Clin Med Phys. 2021 Dec 17;23(3):e13508. doi: 10.1002/acm2.13508 (PMC8906212; doi:10.1002/acm2.13508)
Supplement: Supplementary file 1 — Supporting information [file ACM2-23-e13508-s002.docx]

**Partitioning of ECG-Gated SPECT Raw Data for Protocols Optimization**

**Supplemental Material 1**

The following scripts and comments will guide readers on how to implement the algorithm to input the DICOM archives and obtain the output archives with simulated lower counting statistics. Users must have istalled a Python environment [21], provide the requiring archives, and copy and paste the scripts on the Python IDE to run the algorithm.

**Python script for myocardial SPECT acquisitions:**

**Step 1: Install and import *pydicom* and *numpy* libraries**

**import** **pydicom**

**import** **numpy** **as** **np**

**from** **numpy** **import** *

**Step 2: Load variables (INPUT)**

**Local**: directory to store the output archives.

**dataset_in**: DICOM archive as the input. Please, use a SPECT image with 64 projections and 32, 16 or 8 gated frames.

**dataset_out_perf:** Previously store any SPECT ungated DICOM archive with 64 projections. The DICOM header will be used as the template for the output recombined ungated archive.

**dataset_out_gate**: Previously store any SPECT gated DICOM archive with 64 projections and 8 frames. The DICOM header will be used as the template for the output recombined gated archive.

local='/Users/'

dataset_in=pydicom.dcmread('/Users/DICOM_P64_F32.IMA')

dataset_out_perf=pydicom.dcmread('/Users/DICOM_P64.IMA')

dataset_out_gate=pydicom.dcmread('/Users/DICOM_64_F8.IMA')

**Step 3: Defining the function to recombine the frames and generate four pairs (gated and ungated) of DICOM archives with simulated lower counts (OUTPUT)**

**def** partitioning(dataset_in, dataset_out_perf, dataset_out_gate, local):

ds32 = dataset_in

ds_p = dataset_out_perf

ds_g = dataset_out_gate

data32 = ds32.pixel_array

numProj_32f = ds32.NumberOfFrames

numProj_perf = ds_p.NumberOfFrames

numProj_gate = ds_g.NumberOfFrames

nf32 = ds32.NumberOfTimeSlots

nf8 = ds_g.NumberOfTimeSlots

n_files = int(ds32.NumberOfTimeSlots / ds_g.NumberOfTimeSlots)

lis = []

**for** j **in** range(n_files):

q = list(range(j+1,nf32+1,int(n_files)))

lis.append(q)

aux = zeros((int(n_files)), dtype=int16)-1

row = ds32.Rows

col = ds32.Columns

rD_p = []

rD_g = []

rDperf = zeros((numProj_perf,row,col), dtype=int16)

rDgate = zeros((numProj_gate,row,col), dtype=int16)

**for** n1 **in** range(n_files):

rD_p.append(zeros((numProj_perf,row,col), dtype=int16))

rD_g.append(zeros((numProj_gate,row,col), dtype=int16))

**for** i **in** range(int(ds32.NumberOfFrames)):

a = ds32.DetectorVector[i]

b = ds32.AngularViewVector[i]

c = ds32.TimeSlotVector[i]

d = (a-1)*32+b-1

**for** k **in** range(int(n_files)):

**if** c **in** (lis[k]):

aux[k] = aux[k]+1

rD_p[k][d,:,:] = rD_p[k][d,:,:] + data32[i,:,:]

rD_g[k][aux[k],:,:] = rD_g[k][aux[k],:,:] + data32[i,:,:]

**for** x **in** range(4):

xx = x+1

rDperf = rDperf + rD_p[x]

rDgate = rDgate + rD_g[x]

ds_p.PatientName = "Stat_" + str(xx) + dataset_in.StationName

ds_p.PatientID = "Stat_" + str(xx) + dataset_in.StationName

ds_p.StationName = dataset_in.StationName

ds_p.PixelData = rDperf.tostring()

ds_p.save_as(local + str(xx) + dataset_in.StationName + 'test_phantom_cardiac_.IMA')

ds_g.PatientName= "Stat_" + str(xx) + dataset_in.StationName

ds_g.PatientID= "Stat_" + str(xx) + dataset_in.StationName

ds_g.StationName = dataset_in.StationName

ds_g.PixelData=rDgate.tostring()

ds_g.save_as(local + str(xx) + dataset_in.StationName + 'test_phantom_cardiac_GATE.IMA')

**Step 4: Using the INPUTS, run the function defined in step 3**

partitioning(dataset_in,dataset_out_perf,dataset_out_gate,local)

**##########################################################################################################################################################################################################################################**

**Python script for SPECT acquisition other than myocardial:**

**Step 1: Install and import *pydicom* and *numpy* libraries**

**import** **pydicom**

**import** **numpy** **as** **np**

**from** **numpy** **import** *

**Step 2: Load variables (INPUT)**

**Local**: directory to store the output archives.

**dataset_in**: DICOM archive as the input. Please, use a SPECT image with 64 projections and 32, 16 or 8 gated frames.

**dataset_out:** Previously store any SPECT ungated DICOM archive with 64 projections which DICOM header will be used as the template for the output recombined ungated archive.

local='/Users/'

dataset_in=pydicom.dcmread('/Users/DICOM_P64_F32.IMA')

dataset_out=pydicom.dcmread('/Users/DICOM_P64.IMA')

**Passo 3: Defining the function to recombine the frames and generate 1/n DICOM archives with simulated lower counts (OUTPUT)**

**def** partitioning_other(dataset_in, dataset_out, local):

ds32 = dataset_in

ds_p = dataset_out

data32 = ds32.pixel_array

numProj_32f = ds32.NumberOfFrames

numProj_perf = ds_p.NumberOfFrames

nf32 = ds32.NumberOfTimeSlots

nfrot = ds32.RotationInformationSequence[0][0x0054, 0x0053].value

n_files = int(ds32.NumberOfTimeSlots)

lis = []

**for** j **in** range(n_files):

q = list(range(j+1,nf32+1,int(n_files)))

lis.append(q)

row = ds32.Rows

col = ds32.Columns

rD_p = []

rDperf = zeros((numProj_perf,row,col), dtype=int16)

**for** n1 **in** range(n_files):

rD_p.append(zeros((numProj_perf,row,col), dtype=int16))

**for** i **in** range(int(ds32.NumberOfFrames)):

a = ds32.DetectorVector[i]

b = ds32.AngularViewVector[i]

c = ds32.TimeSlotVector[i]

d = (a-1)*nfrot+b-1

**for** k **in** range(int(n_files)):

**if** c **in** (lis[k]):

rD_p[k][d,:,:] = rD_p[k][d,:,:] + data32[i,:,:]

print('a = ', a, 'b = ', b, 'c = ', c, 'd = ', d)

print('k = ', k, 'lis[k] = ', lis[k])

**for** x **in** range(n_files):

xx = x+1

rDperf = rDperf + rD_p[x]

ds_p.PatientName = "Stat_" + str(xx) + '_' + str(n_files) + dataset_in.StationName

ds_p.PatientID = "Stat_" + str(xx) + '_' + str(n_files) + dataset_in.StationName

ds_p.StationName = dataset_in.StationName

ds_p.PixelData = rDperf.tostring()

ds_p.save_as(local + "Stat_" + str(xx) + '_' + str(n_files) + dataset_in.StationName + '_SPECT.IMA')

**Step 4: Using the INPUTS, run the function defined in step 3**

Partitioning_other(dataset_in,dataset_out,local)
