# Supplementary material for: Technical note: Partitioning of gated single photon emission computed tomography raw data for protocols optimization
Source: J Appl Clin Med Phys. 2021 Dec 17;23(3):e13508. doi: 10.1002/acm2.13508 (PMC8906212; doi:10.1002/acm2.13508)
Supplement: Supplementary file 2 — Supporting information [file ACM2-23-e13508-s001.docx]

**Partitioning of ECG-Gated SPECT Raw Data for Protocols Optimization**

**Supplemental Material 2**

**Phantom preparation, image acquisition and reconstruction:**

The static cardiac phantom ECT/TOR/P was prepared according to the following: the thorax compartment and the cavities that simulates the left ventrical chamber, left ventrical wall and the liver were filled with Tc-99m solution with concentration of 7.6 ($\pm$5%), 7.6 ($\pm$5%), 159 ($\pm$5%) and 94 ($\pm$5%) kBq/ml respectively [22]. The insert that simulates the lesion was filed with water. The 76-825 phantom does not have the liver neither the cardiac lesion chambers. All other compartments were filled with the same activity concentrations as the ECT/TOR/P phantom.

The images were acquired with 64 tomographic projections, step-and-shoot with auto-contour acquisition mode, low energy high-resolution collimators, 140 keV ± 15% Energy, 32 cardiac frames, 20 seconds at each projection. The acquisition matrix was 64x64 and zoom = 1.45 for the e.cam and Symbia Evo systems; 128x128 and zoom = 1.0 for the Symbia Intevo. Mean counts per projection was 114,711 (range: 72,393-153,602), 102,320 (range: 65,670-138,664) and 66,907 (range: 52,200-73,803) for e.cam, Symbia Intevo and Symbia Evo systems, respectively.

The gated raw data was partitioned and recombined using the proposed algorithm to simulate counting levels with 100%, 75%, 50% and 25% of the ungated image. The raw data was analysed by summing projections to measure the counting output. The figure bellow presents the total counts at each projection for simulated countings 100%, 75%, 50% and 25%, in colors black, green, blue and red, respectively.


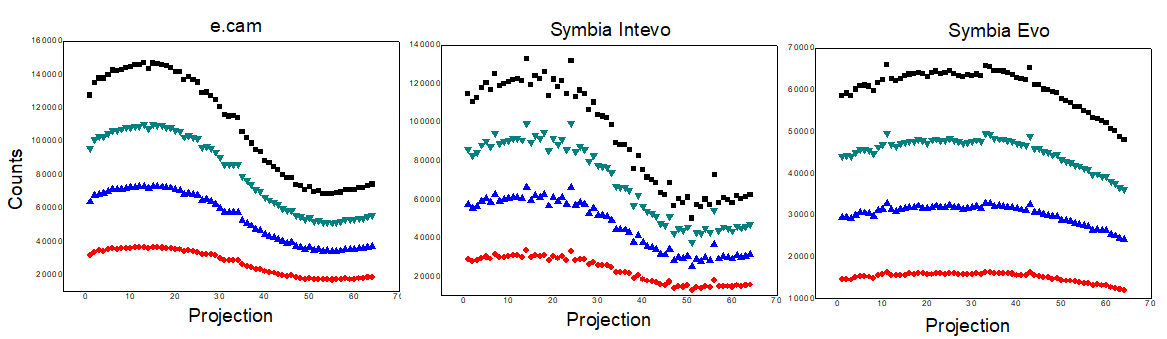


All images were reconstructed with Flash3D^TM^ algorithm (8 iterations, 8 subsets and 14 mm Gausian filter) using a 64x64 matrix (6.6 mm/pixel).
